# On the northernmost *Orchestina* species in China, with a first description of the female of *O. zhiwui* (Araneae, Oonopidae)

**DOI:** 10.3897/zookeys.1022.62387

**Published:** 2021-03-08

**Authors:** Ying Wang, Na Wan, Yanfeng Tong, Yuri M. Marusik

**Affiliations:** 1 College of Life Sciences, Shenyang Normal University, Shenyang 110034, China Shenyang Normal University Shenyang China; 2 Institute for Biological Problems of the North RAS, Portovaya Str. 18, Magadan, Russia Institute for Biological Problems of the North RAS Magadan Russia; 3 Department of Zoology & Entomology, University of the Free State, Bloemfontein 9300, South Africa University of the Free State Bloemfontein South Africa; 4 Zoological Museum, Biodiversity Unit, FI-20014 University of Turku, Turku, Finland University of Turku Turku Finland

**Keywords:** Fenghuang Mountain, Goblin spiders, Key to species, Taxonomy

## Abstract

*Orchestina zhiwui* Liu, Xu & Henrard, 2019, a species previously known only from males collected in Jiangxi Province, was found in Liaoning, ca 2200 km northeast of the type locality, including specimens of both sexes. The previously unknown female of this species is described, and the male is redescribed. A key to species of the genus *Orchestina* from China is provided.

## Introduction

*Orchestina* Simon, 1882 is among the most speciose genera of the goblin spider family (Oonopidae), with 162 extant and 33 fossil species (WSC 2021). It has an almost global distribution and occurs in the Northern Hemisphere in the region south of 45°N (Marusik et al. 2018). In China, the genus is known from 13 species (Tong and Li 2011, 2014; Liu et al. 2016, 2019), and the northernmost localities were previously known from Zhejiang Province (Fig. 4; Li and Lin 2016).

While studying spiders collected on Fenghuang Mountain in the Liaoning Province, China, we found *Orchestina* specimens; this locality is distant from their known range. A detailed study of the males revealed that the specimens belong to *Orchestina zhiwui* Liu, Xu & Henrard, 2019, a species known only from males collected in Jiangxi. The goals of our paper are to provide a key and distribution map to all species of *Orchestina* occurring in China, redescribe the male and provide the first description of the female *O. zhiwui* with detailed illustrations for both sexes.

## Material and methods

The specimens were examined using a Leica M205C stereomicroscope. Details of body parts and measurements were studied under an Olympus BX51 compound microscope. Photos were made with a Canon EOS 750D zoom digital camera (18 megapixels) mounted on an Olympus BX51 compound microscope. Vulvae were cleared in lactic acid. For scanning electron microscopy (**SEM**), specimens were air-dried, sputter coated using IXRF SYSTEMS, and imaged with a Hitachi TM3030 SEM. Photos were stacked using Helicon Focus 7.6.1 and processed using Adobe Photoshop 21.1.2. All measurements in the text are expressed in millimeters. Terminology and taxonomic descriptions follow Henrard and Jocqué (2012) and Tong and Li (2011). All material studied is deposited in Shenyang Normal University (**SYNU**) in Shenyang, China.

The following abbreviations are used in the text and figures: **ALE** = anterior lateral eyes; **ARe** = anterior receptaculum; **AUS** = anterior uterine sclerite; **Mp** = median projection of clypeus; **PLE** = posterior lateral eyes; **PME** = posterior median eyes; **Po** = pore-like structure; **Pp** = posterior plate; **Pr** = protrusions; **Se** = serrula; **So** = slit organs; **Ss** = stomate-like structure; **To** = triangular outgrowth.

## Taxonomy

**Family Oonopidae Simon, 1890**

**Genus *Orchestina* Simon, 1882**

### Key to *Orchestina* species from China

Males of *O. colubrina*, *O. yinggezui*, and *O. zhengi* unknown; female of *O. multipunctata* unknown.

**Table:** 

1 (0)	Males	**2**
–	Females	**11**
2 (1)	Carapace without any pattern	**3**
–	Carapace with reticulate pattern	**5**
3 (2)	Endites without serrula	***O. sinensis* Xu, 1987**
–	Endites with serrula	**4**
4 (3)	Sclerotized part of endites smoothly curved; labium with a sclerotized, inverted Y-shaped pattern; sperm duct with 5 loops in prolateral view (Liu et al. 2019: figs 6B, F, 7A)	***O. bialata* Liu, Xiao & Xu, 2016**
–	Sclerotized part of endites straight; labium without Y-shaped pattern; sperm duct with 3 loops in prolateral view (Tong and Li 2011: figs 2A, 6A)	***O. aureola* Tong & Li, 2011**
5 (2)	Bulb globular; embolus conical, with ventrally swollen base (e.g., Fig. 1K)	**6**
–	Bulb pear-shaped (e.g., Tong and Li 2011: fig. 8A) or globular, but with distal part leading to tube-shaped embolus (e.g., Tong and Li 2011: fig. 9C)	**8**
6 (5)	Palpal tibia distinctly wider than bulb (e.g. Fig. 2E); sperm duct with 1 or 3 loops in prolateral view	**7**
–	Palpal tibia narrower than bulb; sperm duct with 2 loops in prolateral view (Xu 1987: fig. 11)	***O. thoracica* Xu, 1987**
7 (6)	Clypeus with a median projection (Fig. 2H); endites with sub-apical triangular outgrowths (Figs 1H, 2A); sperm duct with 1 loop in prolateral view (Fig. 1I)	***O. zhiwui* Liu, Xu & Henrard, 2019**
–	Clypeus without a median projection; endites without outgrowths (Tong and Li 2011: figs 2B, 7D); sperm duct with 3 loops in prolateral view (Tong and Li 2011: fig. 7A)	***O. clavulata* Tong & Li, 2011**
8 (5)	Bulb globular	**9**
–	Bulb pear-shaped	**10**
9 (8)	Embolus distinctly longer than bulbus; endites with serrula, without modified setae (Liu et al. 2016: figs 1E, 2A, B, 3D, E, 4C, D)	***O. apiculata* Liu, Xiao & Xu, 2016**
–	Embolus shorter than bulbus; endites without serrula, with 2–3 strong setae on anterior margin (Tong and Li 2011: figs 2D, 9C)	***O. tubulata* Tong & Li, 2011**
10 (8)	Chelicerae with a small apophysis on proximal part; endites unmodified (Tong and Li 2011: figs 2C, 3C, D)	***O. truncatula* Tong & Li, 2011**
–	Chelicerae without a small apophysis on proximal part; endites with sharp, hook-shaped distal extension (Liu et al. 2016: figs 8D, 10B)	***O. multipunctata* Liu, Xiao & Xu, 2016**
11 (1)	Carapace without any pattern	**12**
–	Carapace with reticulate pattern	**14**
12 (11)	Epigaster with an oval mark, posteriorly with 2 nearly parallel, longitudinal deep colored stripes (Xu 1987: fig. 4)	***O. sinensis* Xu, 1987**
–	Epigaster without aforementioned character	**13**
13 (12)	Epigaster without a ventral triangular sclerotized plate; anterior part of cylindrical sclerite of endogyne greatly enlarged (Tong and Li 2011: figs 4A, 5D)	***O. aureola* Tong & Li, 2011**
–	Epigaster with a ventral triangular sclerotized plate; anterior part of cylindrical sclerite of endogyne not enlarged (Liu et al. 2016: fig. 5F, G)	***O. bialata* Liu, Xiao & Xu, 2016**
14 (11)	Epigaster with a transverse cuticular fold (Tong and Li 2011: fig. 5I)	***O. zhengi* Tong & Li, 2011**
–	Epigaster without the aforementioned character	**15**
15 (14)	Epigaster with a tubular sclerite visible through the tegument (Tong and Li 2011: fig. 5F)	***O. tubulata* Tong & Li, 2011**
–	Epigaster without the aforementioned character	**16**
16 (15)	Epigaster with large, reddish or dark marks (e.g. Fig. 3E); endogyne with a medial cylindrical sclerite (e.g. Fig. 3F)	**17**
–	Epigaster without large, reddish or dark marks; endogyne with a circular rather than a cylindrical sclerite (e.g. Tong and Li 2011: figs 4D, 5E)	**20**
17 (16)	Abdomen with 3 circumflex-shaped marks	***O. thoracica* Xu, 1987**
–	Abdomen with only 1 circumflex-shaped mark	**18**
18 (17)	Cylindrical sclerite of endogyne greatly enlarged distally (Tong and Li 2011: fig. 5D)	***O. clavulata* Tong & Li, 2011**
–	Cylindrical sclerite of endogyne not enlarged distally	**19**
19 (18)	Cylindrical sclerite encircled medially by tubular sclerite (Fig. 3F)	***O. zhiwui* Liu, Xu & Henrard, 2019**
–	Cylindrical sclerite without the aforementioned character (Liu et al. 2016: fig. 2D)	***O. apiculata* Liu, Xiao & Xu, 2016**
20 (16)	Median part of the epigastric furrow with a vaulted, transverse opening (Tong and Li 2011: figs 4E, 10A)	***O. yinggezui* Tong & Li, 2011**
–	Epigastric furrow without the aforementioned character	**21**
21 (20)	Endogyne with an elongated anterior sclerite (Liu et al. 2019: fig. 10G)	***O. colubrina* Liu, Henrard & Xu, 2019**
–	Endogyne with a very small, chestnut-shaped anterior sclerite (Tong and Li 2011: fig. 5E)	***O. truncatula* Tong & Li, 2011**

#### Orchestina zhiwui

Taxon classificationAnimaliaAraneaeOonopidae

Liu, Xu & Henrard, 2019

CB94D555-F65C-5874-A76B-2AC9640B4CD3

**Figure 1.**
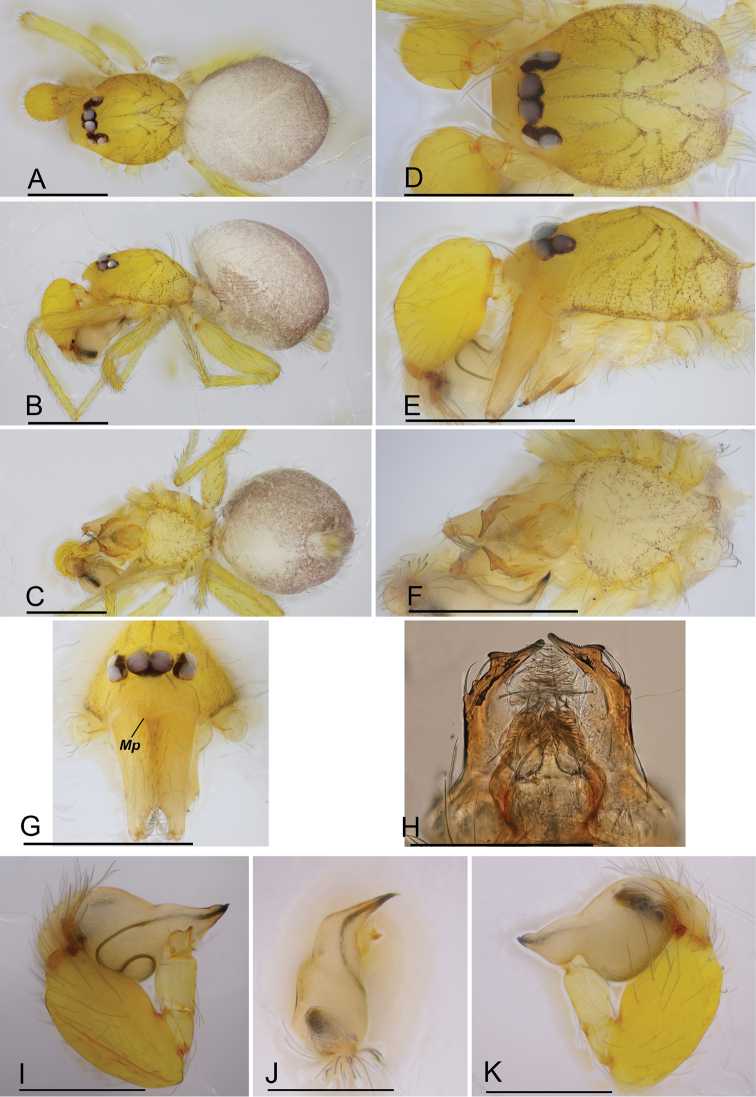
*Orchestina zhiwui* Liu, Xu & Henrard, 2019, male (SYNU-327) **A–C** habitus, dorsal, lateral and ventral views **D–G** prosoma, dorsal, lateral, ventral and anterior views **H** endites and labium, ventral view **I–K** left palp, prolateral, dorsal and retrolateral views. Abbreviations: Mp = median projection of clypeus. Scale bars: 0.4 mm (**A–G**); 0.2 mm (**H–K**).

**Figure 2.**
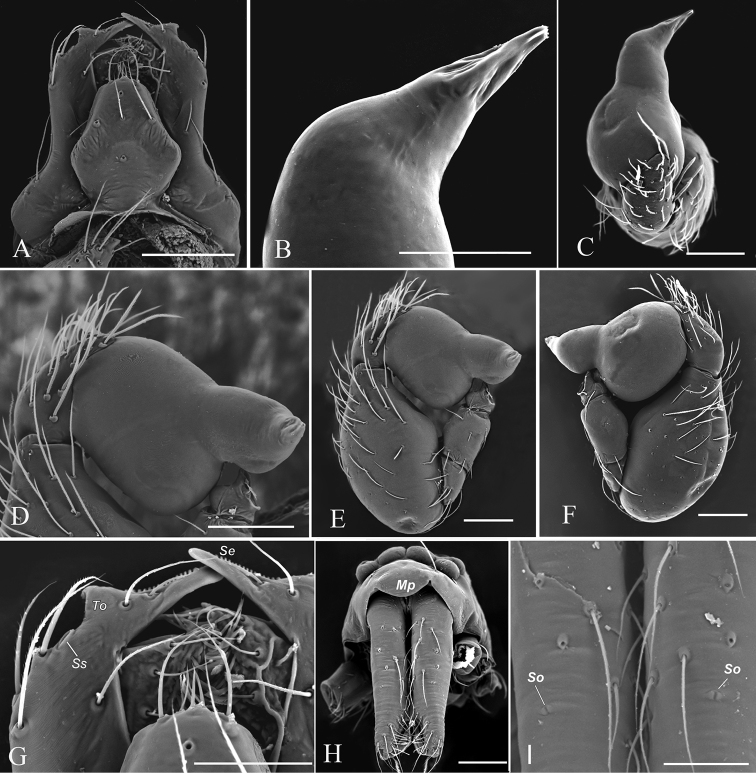
*Orchestina zhiwui* Liu, Xu & Henrard, 2019, male (SYNU-327), SEM**A** labium and endites, ventral views **B** distal part of palpal bulb, dorsal view **C, E, F** left palp, dorsal, prolateral and retrolateral views **D** palpal bulb, prolateral view **G** endites, ventral view **H** prosoma, anterior view **I** detail of chelicerae, highlighting slit organs. Abbreviations: Mp = median projection of clypeus; Se = serrula; So = slit organs; Ss = stomate-like structure; To = triangular outgrowth. Scale bars: 0.1 mm (**A, C, D–F, H, I**); 0.05 mm (**B, G**).

**Figure 3.**
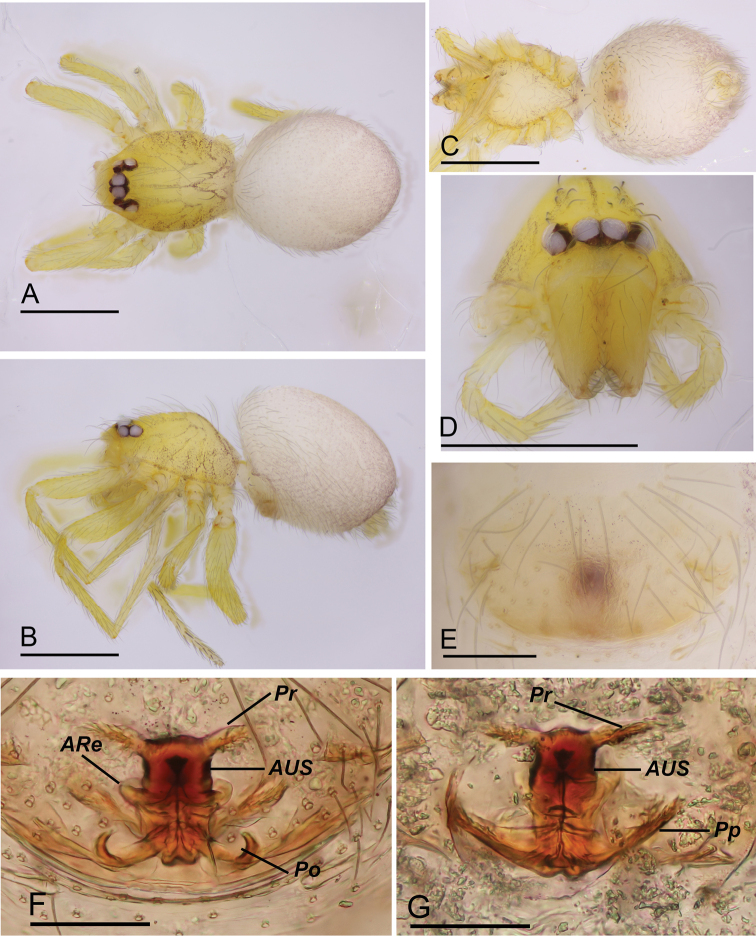
*Orchestina zhiwui* Liu, Xu & Henrard, 2019, female (SYNU-332) **A–C** habitus, dorsal, lateral and ventral views **D** prosoma, anterior view **E** epigaster, ventral view **F, G** endogyne, ventral and dorsal views. Abbreviations: ARe = anterior receptaculum; AUS = anterior uterine sclerite; Po = pore-like structure; Pp = posterior plate; Pr = protrusions. Scale bars: 0.4 mm (**A–D**); 0.1 mm (**E–G**).

**Figure 4.**
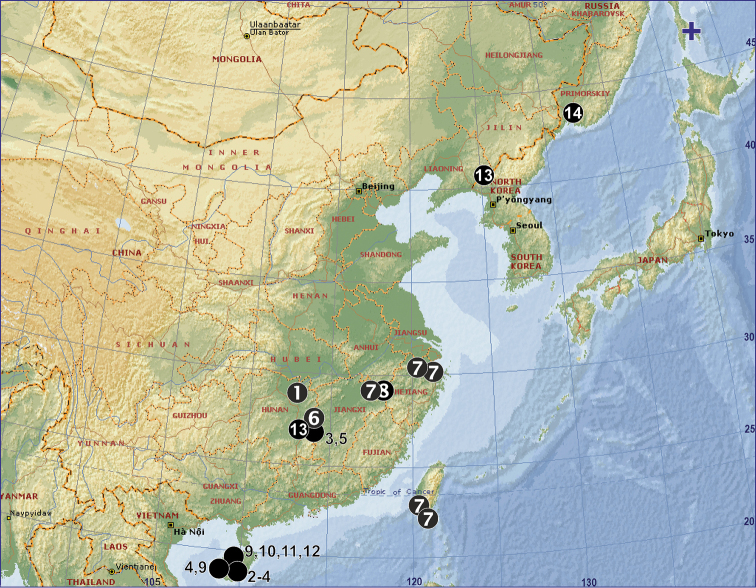
Distribution records of *Orchestina* species from China and the northernmost species in Asia. 1. *O. apiculata* Liu, Xiao & Xu, 2016; 2. *O. aureola* Tong & Li, 2011; 3. *O. bialata* Liu, Xiao & Xu, 2016; 4. *O. clavulata* Tong & Li, 2011; 5. *O. colubrina* Liu, Henrard & Xu, 2019; 6. *O. multipunctata* Liu, Xiao & Xu, 2016; 7. *O. sinensis* Xu, 1987; 8. *O. thoracica* Xu, 1987; 9. *O. truncatula* Tong & Li, 2011; 10. *O. tubulata* Tong & Li, 2011; 11. *O. yinggezui* Tong & Li, 2011; 12. *O. zhengi* Tong & Li, 2011; 13. *O. zhiwui* Liu, Xu & Henrard, 2019; 14. *O. storozhenkoi* (Saaristo & Marusik, 2004); +. *O. sakhalinensis* Marusik, Perkovsky & Eskov, 2018.

Figures 1 , 2 , 3 , 4

O. zhiwui Liu, Xu & Henrard in Liu et al. 2019: 250, figs 12A–I, 13A–C, 14A–I, 15A–G.

##### Material examined.

1♂: China, Liaoning Province, Fengcheng City, Fenghuang Mountain, Cuijiapuzi Village, sifting leaf litter; 26°24'35"N, 124°3'7"E, 130 m; 10.X.2020; Weihua Cheng, Ying Huang, Xiaochen Sun & Yanfeng Tong leg. (SYNU-327); 1♀: same data as previous (SYNU-332); 4♂, same data as previous (SYNU-328-329-330-331); 4♀: same data as previous (SYNU-333-334-335-336); 1♀: same locality; 18.X. 2017; Y.M. Marusik & Bingchuan Zhang leg. (SYNU-337).

##### Diagnosis.

This species is similar to *O. aureola* Tong & Li, 2011 in the shape of the bulb and the short embolus, but can be distinguished by the reticulate carapace pattern (Figs 1A, 3A) in both sexes (vs. uniformly colored (Tong and Li 2011: fig. 1A)). Males can further be recognized by the median projection (Figs 1G, 2H) of the clypeus (vs. with straight anterior margin (Tong and Li 2011: fig. 3A)), the endites with sub-apical triangular outgrowths (Figs 1H, 2A, G) (vs. outgrowths absent (Tong and Li 2011: fig. 2A)) and short sperm duct forming one coil (Fig. 1I) (vs. with several coils (Tong and Li 2011: fig. 6A)). Females can be distinguished by the presence of the tubular sclerite (Fig. 3F) of the endogyne (vs. tubular sclerite absent (Tong and Li 2011: fig. 5A)).

##### Redescription of male

**(SYNU-327). *Body***: habitus as in Fig. 1A–C; body length 1.29. ***Carapace*** (Fig. 1D, E, G): 0.61 long, 0.44 wide; yellow, oval in dorsal view, surface smooth, with net-shaped pattern, with sparse long setae, pars cephalica slightly elevated in lateral view, anterior margin straight in dorsal view, posterolateral corners rounded. ***Eyes*** (Fig. 1D, G): well-developed, PME largest; posterior eye row recurved from above; ALE-PLE touching, PLE-PME separated by less than PME radius, PME touching throughout most of their length. ***Clypeus*** (Figs 1D, E, G, 2H): with a median projection (Mp) in frontal view, sloping forward in lateral view, high, ALE separated from edge of carapace by 1.7 times their diameter; with pairs of long needle-like setae in front of ALE. ***Sternum*** (Fig. 1F): longer than wide, yellow, with scattered sepia pigmentation, surface smooth, without radial furrows between coxae; setae sparse, needle-like, evenly scattered, without hair tufts. ***Mouthparts*** (Figs 1F–H, 2A, G–I): chelicerae straight and long, 4 times longer than wide, with eye-shaped slit organ (So) in medial part; labium as an elongated hexagon, anterior margin not indented at middle; endites strongly sclerotized, except mesal part, basally with shallow diagonal furrow, with elongated extension bearing serrula (Se), sub-apical triangular outgrowth (To) and stomate-like structure (Ss). ***Abdomen*** (Fig. 1A–C): 0.74 long; grayish, with a pale narrow chevron. ***Legs***: yellow, femur IV thickened, wider than femora I–III, without spines. ***Palp*** (Figs 1I–K, 2B–F): tibia enlarged and strongly swollen, 1.6 times longer than wide and 2 times longer than femur plus patella; cymbium ovoid; bulb stout, basal part globular, wider than tibia width, with ventral boss proximally; embolus short, conical, with ventrally swollen base; sperm duct with 1 loop on prolateral side, opening of sperm duct small, round, located on tip.

##### Description of female

**(SYNU-332).** Same as male except as noted. ***Body***: habitus as in Fig. 3A–C; body length 1.26. ***Carapace***: 0.59 long, 0.42 wide. ***Clypeus*** (Fig. 3D): anterior margin straight. ***Mouthparts***: chelicerae shorter; endites simple, with serrula. ***Abdomen***: 0.69 long. ***Epigaster*** (Fig. 3E, F): without special external features; internal parts visible through integument. ***Endogyne*** (Fig. 3G): with medial cylindrical sclerite (AUS), encircled medially by tubular sclerite corresponding to anterior receptaculum (ARe), anterior part of cylindrical sclerite (AUS) with pair of lateral protrusions (Pr); posterior part with pair of pore-like structures (Po) on ventral side and posterior plate (Pp) on dorsal side.

##### Habitats.

All specimens were collected in leaf litter in a mountain forest.

##### Distribution.

China (Liaoning and Jiangxi) (Fig. 4).

## Discussion

### Morphology

While studying the morphology of *O. zhiwui*, we found a character that is undocumented in other *Orchestina* species: slit organs anteromedially on the chelicerae of the male (Fig. 2I). The chelicerae of many *Orchestina* species were quite well illustrated by Henrard and Jocqué (2012) and Izquierdo and Ramírez (2017), but this character does not appear in any descriptions or images. Possibly this character is related to the very long chelicera of the male.

### Distribution

Although the record from Liaoning is the northernmost record of this species in China and extends its known distribution limits over 1200 km to the northeast (see Fig. 4), it is not the northernmost species of the genus in Asia. That record belongs to *O. storozhenkoi* (Saaristo & Marusik, 2004) described from the Maritime Province of Russia. It was described in a monotypic genus, *Ferchestina* Saaristo & Marusik, 2004, which was later synonymized by Platnick et al. (2012). This species is found on tree trunks (Saaristo and Marusik 2004) rather than in leaf litter like *O. zhiwui*. There is at least one more northern record of *Orchestina*, *O. sakhalinensis* Marusik, Perkovsky & Eskov, 2018, but it belongs to a fossil species from Sakhalin Island. This species was found in amber deposits near Starodubskoye, ca. 47°24'N (Marusik et al. 2018).

## Supplementary Material

XML Treatment for Orchestina zhiwui
